# Assessment of lung function in successfully treated tuberculosis reveals high burden of ventilatory defects and COPD

**DOI:** 10.1371/journal.pone.0217289

**Published:** 2019-05-23

**Authors:** Akshay N. Gupte, Mandar Paradkar, Sriram Selvaraju, Kannan Thiruvengadam, Shri Vijay Bala Yogendra Shivakumar, Krithikaa Sekar, Srinivasa Marinaik, Ayesha Momin, Archana Gaikwad, Premkumar Natrajan, Munivardhan Prithivi, Gomathy Shivaramakrishnan, Neeta Pradhan, Rewa Kohli, Swapnil Raskar, Divyashri Jain, Rani Velu, Bharath Karthavarayan, Rahul Lokhande, Nishi Suryavanshi, Nikhil Gupte, Lakshmi Murali, Sundeep Salvi, William Checkley, Jonathan Golub, Robert Bollinger, Vidya Mave, Chandrasekaran Padmapriyadarasini, Amita Gupta

**Affiliations:** 1 Johns Hopkins University School of Medicine, Baltimore, United States of America; 2 Byramjee Jeejeebhoy Government Medical College-Johns Hopkins University Clinical Research Site, Pune, India; 3 National Institute for Research in Tuberculosis, Chennai, India; 4 Johns Hopkins University India Office, Pune, India; 5 Byramjee Jeejeebhoy Government Medical College and Sassoon General Hospitals, Pune, India; 6 Chest Research Foundation, Pune, India; Jamia Hamdard, INDIA

## Abstract

**Background:**

Burden, phenotype and risk-factors of lung function defects in successfully treated tuberculosis cases are unclear.

**Methods:**

We performed spirometry with bronchodilators in new drug-sensitive adult (≥18 years) pulmonary tuberculosis cases during the 12 months following successful treatment in India. Airflow obstruction was defined as pre-bronchodilator FEV1/FVC<5^th^ percentile of Global Lung Initiative mixed-ethnicity reference (lower limit of normal [LLN]). Chronic obstructive pulmonary disease (COPD) was defined as post-bronchodilator FEV1/FVC<LLN among participants with obstruction. Restrictive spirometry pattern was defined as FVC<LLN among participants without obstruction. Multivariable logistic and linear regression was used to identify risk-factors for obstruction, restriction and low lung function despite successful treatment.

**Results:**

Of the 172 participants included in the analysis, 82 (48%) were female, 22 (13%) had diabetes and 34 (20%) ever-smoked with a median (IQR) exposure of 3.5 (0.2–9.9) pack-years. Median (IQR) age and body-mass index (BMI) at enrollment was 32 (23–39) years and 18.1 (16.0–20.5) kg/m^2^ respectively. Airflow obstruction was detected in 42 (24%) participants; of whom 9 (21%) responded to short-acting bronchodilators and 25 (56%) had COPD; and was associated with duration of illness prior to treatment (aOR = 1.32 per 30-days, 95%CI 1.04–1.68, p = 0.02). A restrictive spirometry pattern was detected in 89 (52%) participants and was associated with female sex (aOR = 3.73, 95%CI 1.51–9.17, p = 0.004) and diabetes (aOR = 4.06, 95%CI 1.14–14.42, p = 0.03). Higher HbA1c at treatment initiation was associated with greater odds of a restrictive spirometry pattern (aOR = 1.29 per unit higher HbA1c, 95%CI 1.04 to 1.60, p = 0.02).

**Conclusion:**

We found a high burden of lung function defects and COPD in tuberculosis cases who successfully completed treatment. Screening for chronic lung diseases following treatment and linkage to respiratory health clinics should be included in the routine management plan of all tuberculosis cases in India, regardless of conventional COPD risk-factors such as older age and smoking.

## Introduction

Tuberculosis disease (TB) is the leading infectious killer worldwide with over 10 million incident cases and 1.5 million deaths in 2017[1]. Pulmonary TB, the most common form of the disease, is curable with multi-drug therapy and most high burden countries report cure rates exceeding 80%[1]. However, microbiological cure may not prevent pulmonary complications of TB and there is increasing evidence to suggest lung injury can persist despite TB treatment, leading to chronic pulmonary sequelae and disability[2–5].

With an estimated 174.5 million prevalent cases and over 3.2 million deaths in 2016, chronic obstructive pulmonary disease (COPD) is the third-leading cause of death globally[6, 7]. While smoking remains the key risk factor for COPD, a considerable burden of the disease in low- and middle-income countries cannot be explained by smoking alone; TB and other non-smoking risk-factors of COPD are gaining importance[8]. Cross-sectional studies have found a higher prevalence of COPD in individuals with a self-reported history of TB[9–13], and a recent meta-analysis found a 3-fold higher odds of airflow obstruction in individuals with prior TB[14]. The intersection of TB and COPD is an urgent threat to lung health globally and particularly in India. India hosts the world’s largest burden of TB with nearly 3 million new cases and a rapidly growing burden of COPD with an estimated 55.3 million prevalent cases in 2016[1, 15]. Furthermore, COPD was the second-leading non-communicable cause of death and disability accounting for nearly 11% and 7% of all deaths and Disability Adjusted Life Years (DALYs) lost in India in 2016, respectively[16].

Current guidelines for management of TB cases do not recommend screening for chronic lung diseases following treatment. This may be due in part to insufficient evidence as few studies have assessed the burden and phenotype of chronic lung disease in well characterized prospective cohorts of successfully treated TB cases from high-burden settings, especially in young adults with minimal exposure to tobacco smoke who are at low risk of chronic lung diseases independent of TB. Importantly, risk-factors including commonly occurring comorbid conditions like diabetes that may be associated with pulmonary impairment despite microbiological cure of TB are unclear. Addressing these knowledge gaps can help estimate the burden of chronic lung diseases in populations that are typically not considered high-risk due to the absence of conventional risk-factors and, identify TB cases most likely to benefit from targeted monitoring and linkage to appropriate respiratory health clinics for further management.

Therefore, we sought to evaluate the burden, phenotype and risk-factors of chronic lung disease, defined by spirometry, in a prospective cohort of young and predominantly never-smoking adults with pulmonary TB who successfully completed their treatment in India.

## Methods

### Study population and procedures

We enrolled new adult (≥18 years) pulmonary TB cases who successfully completed TB treatment from the ongoing CTRIUMPH study between June 2016 and July 2018. Briefly, the CTRIUMPH study is a prospective cohort study within the RePORT-India TB research consortium that is evaluating new drug-sensitive TB cases presenting to the Revised National TB Control Program clinics at the Byramjee-Jeejeebhoy Government Medical College-Sassoon General Hospitals (BJGMC-SGH) in Pune and the National Institute for Research in Tuberculosis (NIRT) in Chennai, India[17]. Pulmonary TB cases were diagnosed by the presence of acid-fast bacilli (AFB) on smear microscopy, *Mycobacterium tuberculosis* DNA on Xpert MTB/RIF assay, *M*.*tuberculosis* growth on Mycobacterial Growth Indicator Tube (MGIT) liquid culture or Löwenstein -Jensen (LJ) solid culture media, or based on clinical judgement in the absence of microbiological confirmation of TB. Participants with prior TB and those with rifampicin resistance were excluded.

Socio-demographic, clinical and laboratory data were collected using standardized questionnaires and operating procedures. Ever-smokers were classified at TB treatment initiation as individuals who self-reported smoking at least 100 tobacco products during their lifetime. Alcohol use disorder (AUD) was defined as having an Alcohol Use Disorder Identification Test score of at least 8 points[18]. Diabetes was defined at TB treatment initiation as glycated hemoglobin (HbA1c) of at least 6.5% or a prior physician diagnosis. Cavitary disease was identified on chest radiographic evaluation at TB treatment initiation. Duration of illness was calculated by the number of days between self-reported symptom onset and the first dose of standard multi-drug therapy.

Enrolled participants underwent pre- and post-bronchodilator spirometry during a 12-month interval following the last dose of their TB treatment. Spirometry was performed by trained study staff using the EasyOne ultrasonic flow spirometer (ndd Medical, Zurich) in the sitting position. The device was calibrated on the day of a scheduled spirometry evaluation using a standardized 3L calibration syringe. Participants performed a minimum of three and maximum of eight maneuvers according to the American Thoracic Society and European Respiratory Society guidelines[19]. Post-bronchodilator spirometry was performed 15 minutes after administering 200μgms of salbutamol sulphate via capsule based dry-powder inhaler (Revolizer, Cipla Ltd, Mumbai). The EasyOne spirometer has a built-in quality assessment software that automatically generates a quality grade ranging from “A” to “D” and “F”. Grades “A”, “B” and “C” indicate at least two acceptable maneuvers that are reproducible within 200mL and were included in the analysis; maneuvers with grades “D” and “F” were excluded. All spirometry tracings underwent independent quality checks for acceptability by a trained pulmonologist prior to inclusion for analysis.

Respiratory health status was assessed by the Saint George’s Respiratory Questionnaire (SGRQ), a respiratory disease-specific health-related Quality of Life instrument validated for use in pulmonary TB[20]. The total SGRQ score measures an individual’s respiratory health status and ranges from 0 to 100, where a score of 0 indicates optimal respiratory health status and higher scores indicate worse respiratory health status. The minimum clinically important difference in total SGRQ scores has been found to be 4 points[21–23]. Hindi, Marathi and Tamil translated versions of the SGRQ were administered to study participants in their native language by trained study staff along with spirometry evaluations during the 12-month interval following TB treatment completion.

Treatment failure was defined during the last two months of treatment as *M*.*tuberculosis* growth on MGIT or LJ culture regardless of symptoms, or AFB detected on smear microscopy and symptoms suggestive of TB disease in participants with unavailable culture results. Recurrent TB was defined among TB cases who did not fail treatment as *M*.*tuberculosis* growth on MGIT or LJ culture regardless of symptoms, or AFB detected on smear microscopy and symptoms suggestive of TB disease in participants with unavailable culture results. TB cases who completed at least 6 months of treatment and did not experience treatment failure or recurrence were classified as having successfully completed TB treatment.

All study participants signed written informed consent in their local language and the protocol was approved by the Institutional Review Boards of Johns Hopkins School of Medicine, BJGMC-SGH and NIRT.

### Statistical analysis

Participants who failed to successfully complete treatment, including those with recurrent TB, were excluded from the analysis. We additionally excluded individuals with a self-reported history of chronic lung diseases.

We calculated percent-predicted values and z-scores of forced expiratory volume in the first second (FEV1), forced vital capacity (FVC) and FEV1 to FVC ratio (FEV1/FVC) for a given age, sex and height using the Global Lung Function Initiative (GLI) reference equations for mixed-ethnicity[24]. Percent-predicted peak expiratory flow rate (PEFR) was calculated using reference equations by Kodgule and colleagues[25]. Percent-predicted FEV1, FVC and PEFR during the 12-month interval following successful TB treatment was compared by participant characteristics at TB treatment initiation using the Kruskal-Wallis test. We used univariable and multivariable linear regression to measure the association of participant characteristics at TB treatment initiation with the mean difference in z-scores of FEV1, FVC and FVE1/FVC following successful treatment. We classified airflow obstruction (AO) as a pre-bronchodilator FEV1/FVC less than the lower limit of normal (LLN), defined as z-scores less than -1.64 which corresponds to the 5^th^ percentile of the normal FEV1/FVC distribution[26]. Reversibility of AO was defined as FEV1 increase of at least 12% and 200mL following the administration of bronchodilators relative to the pre-bronchodilator value[26]. Participants with AO were further classified as having COPD if the post-bronchodilator FEV1/FVC was less than the 5^th^ percentile (z-score of -1.64) of the normal FEV1/FVC distribution. Among participants without AO, we classified restrictive spirometry pattern (RSP) as a pre-bronchodilator FVC z-score less than -1.64 which corresponds to the 5^th^ percentile of the normal FVC distribution[26]. We additionally conducted a secondary analysis using the fixed-ratio definition for AO; i.e. FEV1/FVC below 70%[27]. We used univariable and multivariable logistic regression to identify participant characteristics at TB treatment initiation associated with AO and RSP following successful treatment. Total SGRQ scores measured at the time of spirometry evaluations were compared by chronic lung disease phenotype using the Kruskal-Wallis test.

Continuous data are presented as medians with inter-quartile range (IQR), or mean with 95% confidence intervals (CI) for regression coefficients. Categorical data are presented as frequencies and proportions. Multivariable analysis included variables known to be associated with pulmonary impairment identified a-priori by literature review and post-hoc by exploratory data analysis. Statistical significance was determined at p<0.05. Analyses were done in Stata V.15.0 (StataCorp, USA). A proportional Venn diagram was created using the “pvenn” package.

## Results

Of the 204 participants enrolled, 172 (84%) were included in the analysis; we excluded 11 (5%) participants who failed treatment, 7 (3%) with recurrent TB and 14 (7%) with poor quality spirometry. Nearly 70% (119 of 172) of participants underwent spirometry evaluations within 6 months of completing treatment. The median (IQR) age at enrollment was 32 (23–39) years and 82 (48%) participants were female. The median (IQR) body mass index (BMI) was 18.1 (16.0–20.5) kg/m^2^ and 87 (51%) participants were underweight (BMI<18.5kg/m^2^). Ever-smoking was reported by 34 (20%) participants with a median (IQR) exposure of 3.5 (0.2–9.9) pack-years. Overall, 113 (66%) participants had culture confirmed TB, 22 (13%) had diabetes, 7 (4%) had HIV and 56 (33%) had cavitary disease at treatment initiation. The median (IQR) duration of illness prior to treatment initiation was 30 (20–60) days. (Table 1).

**Table 1:** Percent-predicted lung function following successful treatment by participant characteristics at treatment initiation.

Characteristics	Full Cohort	Percent-predicted FEV1	Percent-predicted FVC	Percent-predicted PEFR
	n (%)	median (IQR)	median (IQR)	median (IQR)
**Age (years)**				
18–29	72 (42)	69 (54–81)	72 (62–86)	70 (56–88)
30–39	58 (34)	68 (59–84)	71 (63–86)	78 (61–93)
≥ 40	42 (24)	73 (57–81)	76 (65–85)	75 (47–88)
p-value		0.52	0.48	0.31
**Sex**				
Female	82 (48)	67 (58–77)	70 (59–80)	78 (60–91)
Male	90 (52)	74 (57–87)	77 (66–89)	72 (46–90)
p-value		0.02	0.004	0.10
**BMI (kg/m**^**2**^**)**				
>18.5	82 (49)	72 (61–81)	75 (66–83)	82 (59–96)
16–18.5	45 (27)	77 (57–85)	82 (62–94)	73 (48–91)
<16	42 (25)	62 (48–71)	68 (53–74)	63 (53–75)
p-value		0.001	0.003	0.01
**Alcohol use disorder**				
Yes	37 (22)	69 (51–80)	74 (64–85)	57 (44–75)
No	135 (78)	70 (59–82)	72 (64–85)	78 (60–93)
p-value		0.59	0.63	0.003
**Ever-smoking**				
Yes	34 (20)	64 (48–80)	73 (62–85)	56 (41–87)
No	138 (80)	70 (59–82)	74 (64–85)	76 (60–93)
p-value		0.17	0.90	0.006
**Formal education**				
<5 years	36 (21)	67 (51–71)	71 (63–76)	64 (43–88)
5–10 years	75 (44)	72 (59–83)	74 (65–87)	73 (55–88)
≥11 years	61 (35)	70 (58–86)	75 (63–90)	80 (62–97)
p-value		0.08	0.19	0.05
**Duration of illness**				
<30 days	69 (40)	75 (69–87)	77 (71–92)	80 (66–91)
30–89 days	70 (41)	59 (52–76)	65 (55–79)	66 (46–87)
≥90 days	32 (19)	70 (58–80)	74 (66–84)	72 (50–95)
p-value		<0.001	<0.001	0.05
**Cavitation**				
Yes	56 (33)	72 (58–83)	75 (65–85)	72 (53–86)
No	116 (67)	69 (57–81)	72 (63–86)	74 (59–94)
p-value		0.62	0.42	0.13
**Diabetes**				
Yes	22 (13)	72 (59–77)	72 (65–79)	72 (44–88)
No	150 (87)	70 (58–83)	74 (63–87)	74 (56–91)
p-value		0.72	0.37	0.44
**HIV coinfection**				
Yes	7 (4)	78 (39–87)	71 (44–96)	93 (59–111)
No	165 (96)	70 (58–81)	74 (64–85)	73 (56–90)
p-value		0.71	0.71	0.14
**Smear grade**				
Negative	109 (63)	71 (59–83)	74 (65–86)	75 (59–93)
1+	48 (28)	69 (57–82)	74 (61–88)	72 (54–90)
≥2+	15 (9)	67 (44–71)	71 (59–77)	72 (36–82)
p-value		0.19	0.38	0.13
**Culture**				
Positive	113 (67)	69 (57–76)	72 (63–79)	72 (53–88)
Negative	56 (33)	78 (61–89)	79 (62–93)	81 (64–95)
p-value		0.009	0.11	0.01

FEV1—forced expiratory volume in the first second, FVC—forced vital capacity, PEFR—peak expiratory flow rate, IQR—interquartile range, BMI—body mass index.

**Fig 1:**
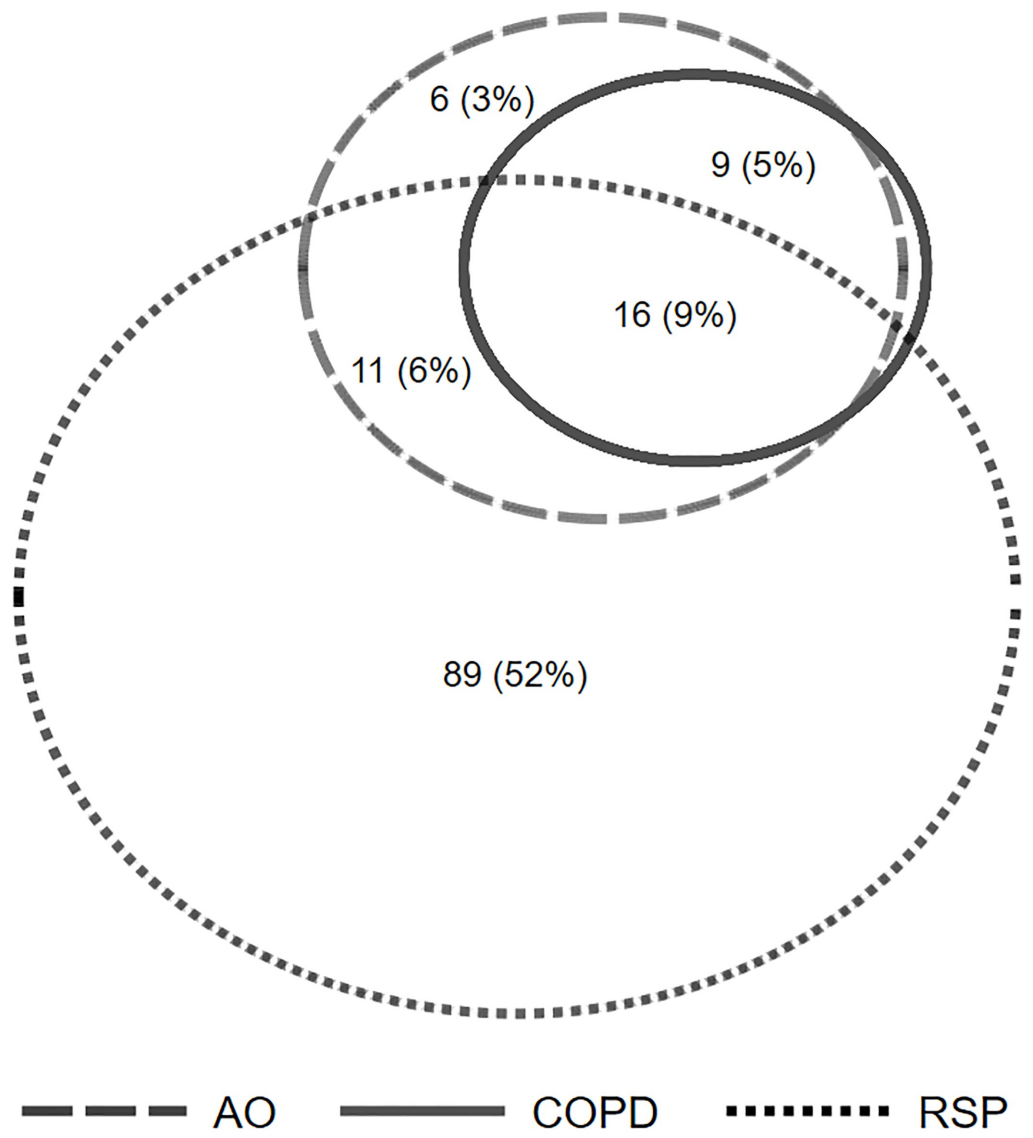
Burden of lung function defects among successfully treated TB cases. AO—airflow obstruction, COPD—chronic obstructive pulmonary disease, RSP—restrictive spirometry pattern. Burden of lung function defects calculated as a proportion of the total sample size (n = 172).

The median (IQR) percent-predicted FEV1, FVC and PEFR following successful TB treatment was 70 (58–82)%, 73 (64–85)% and 74 (56–91)% respectively. AO was detected in 42 (24%) participants, 9 (21%) had a positive bronchodilator response and 25 (56%) had COPD. RSP was detected in 89 (52%) participants without AO. Only 40 (23%) participants who successfully completed TB treatment had normal lung function (Fig 1). As expected, participants with AO had significantly higher SGRQ scores indicating worse respiratory health status compared to those without any ventilatory defects. Among participants with AO, those with a further classification of COPD had worse SGRQ scores, FEV1 and PEFR compared to those with AO but without COPD, however this difference did not reach statistical significance. Similarly, participants with AO and a positive bronchodilator response had significantly worse lung function relative to those who did not respond to short acting bronchodilators. (Table 2).

**Table 2:** Total SGRQ scores and percent-predicted lung function by lung function defect classification following successful treatment.

Classification	Total SGRQ score	Percent-predicted FEV1	Percent-predicted FVC	Percent-predicted PEFR
	median (IQR)	median (IQR)	median (IQR)	median (IQR)
**Lung function defect**				
None	5 (2–9)	90 (84–97)	90 (85–94)	96 (83–104)
RSP	7 (4–18)	69 (59–75)	68 (59–94)	74 (61–88)
AO	12 (4–23)	53 (42–65)	70 (62–74)	45 (36–62)
p-value	0.003	<0.001	<0.001	<0.001
**Obstruction**				
None*	6 (2–15)	74 (64–83)	74 (66–84)	80 (65–95)
AO without COPD	12 (8–23)	59 (44–66)	73 (59–87)	60 (38–66)
COPD	17 (3–23)	49 (39–64)	67 (59–94)	44 (27–54)
p-value	0.02	<0.001	0.77	<0.001
Bronchodilator response**				
Negative	10 (4–22)	59 (49–69)	75 (62–99)	75 (59–91)
Positive	26 (5–31)	42 (25–44)	59 (53–66)	31 (18–36)
p-value	0.30	<0.001	0.01	<0.001

*—includes participants with RSP,

**—analysis restricted to participants with AO. SGRQ—Saint George’s Respiratory Questionnaire, FEV1—forced expiratory volume in the first second, FVC—forced vital capacity, PEFR—peak expiratory flow rate, IQR—inter quartile range, RSP—restrictive spirometry pattern, AO—airflow obstruction, COPD—chronic obstructive pulmonary disease.

**Fig 2:**
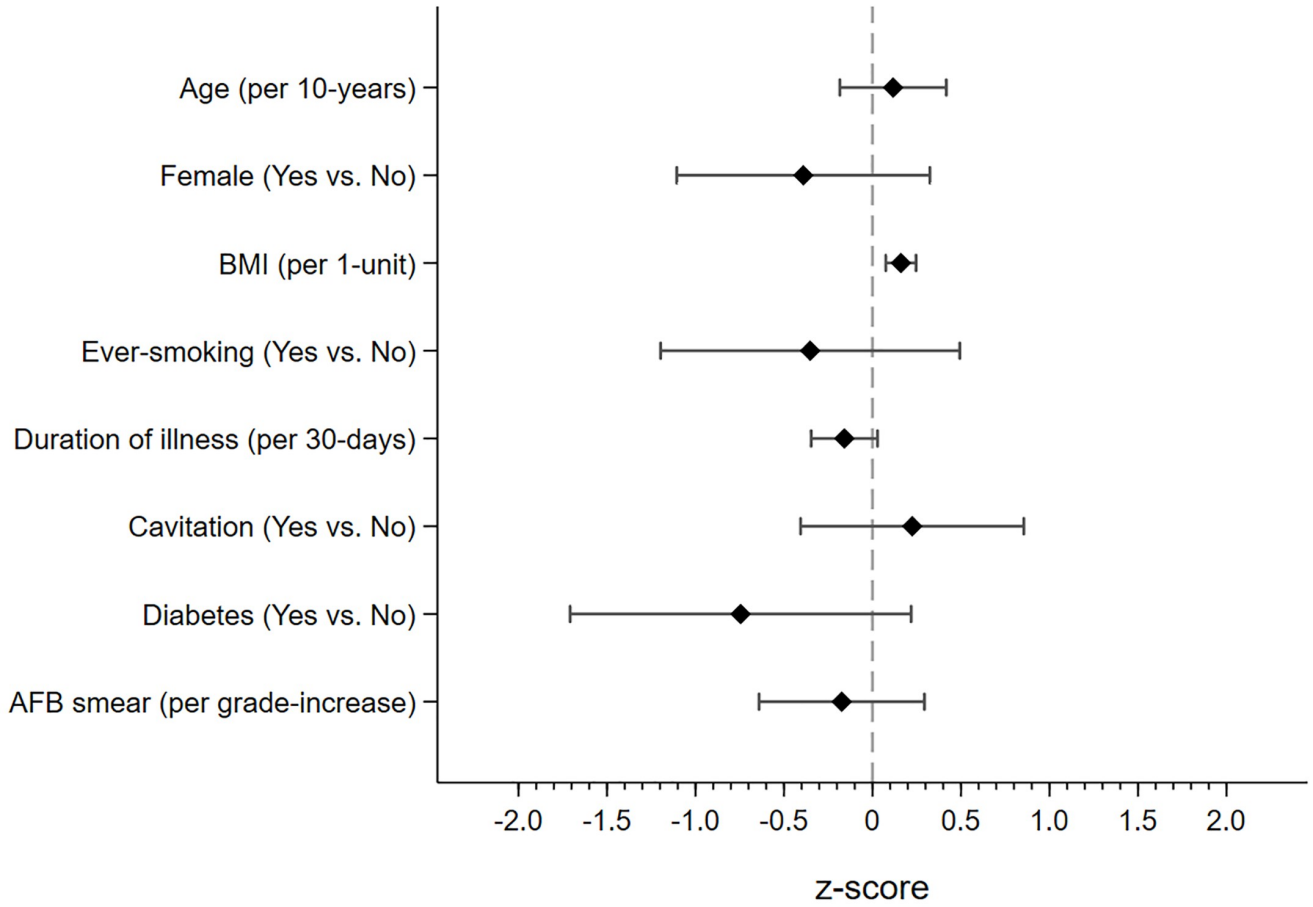
Difference in FEV1 z-score by participant characteristics at treatment initiation. Figure depicts point estimate and accompanying 95% confidence interval for FEV1 standardized by z-scores. Higher z-scores indicate better FEV1. Regression analysis was adjusted for age, sex, BMI, ever-smoking, duration of illness, cavitation, diabetes and smear grade. FEV1 —forced expiratory volume in the first second, BMI—body mass index, AFB—acid fast bacilli.

**Fig 3:**
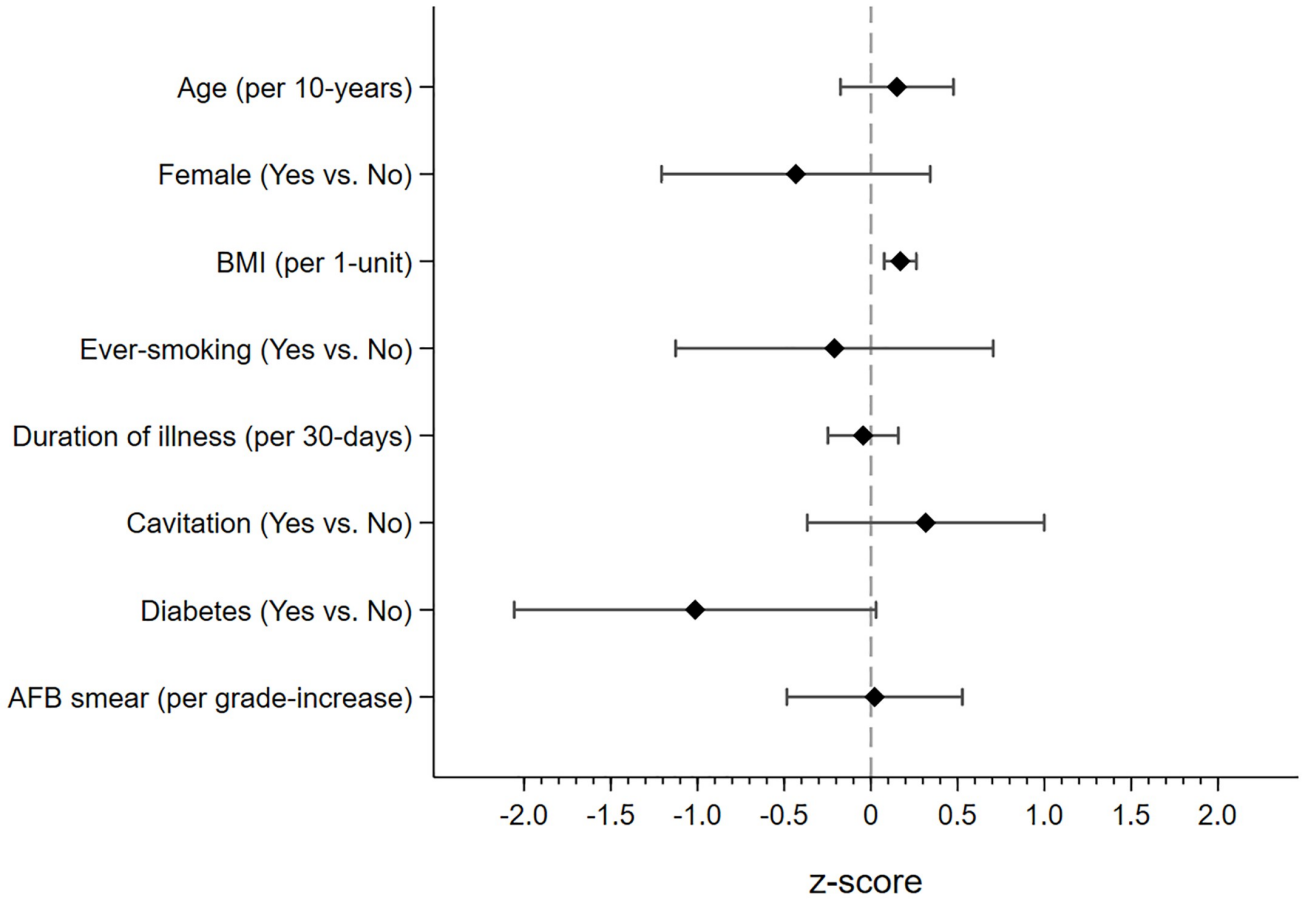
Difference in FVC z-score by participant characteristics at treatment initiation. Figure depicts point estimate and accompanying 95% confidence interval for FVC standardized by z-scores. Higher z-scores indicate better FVC. Regression analysis was adjusted for age, sex, BMI, ever-smoking, duration of illness, cavitation, diabetes and smear grade. FVC—forced expiratory volume, BMI—body mass index, AFB—acid fast bacilli.

**Fig 4:**
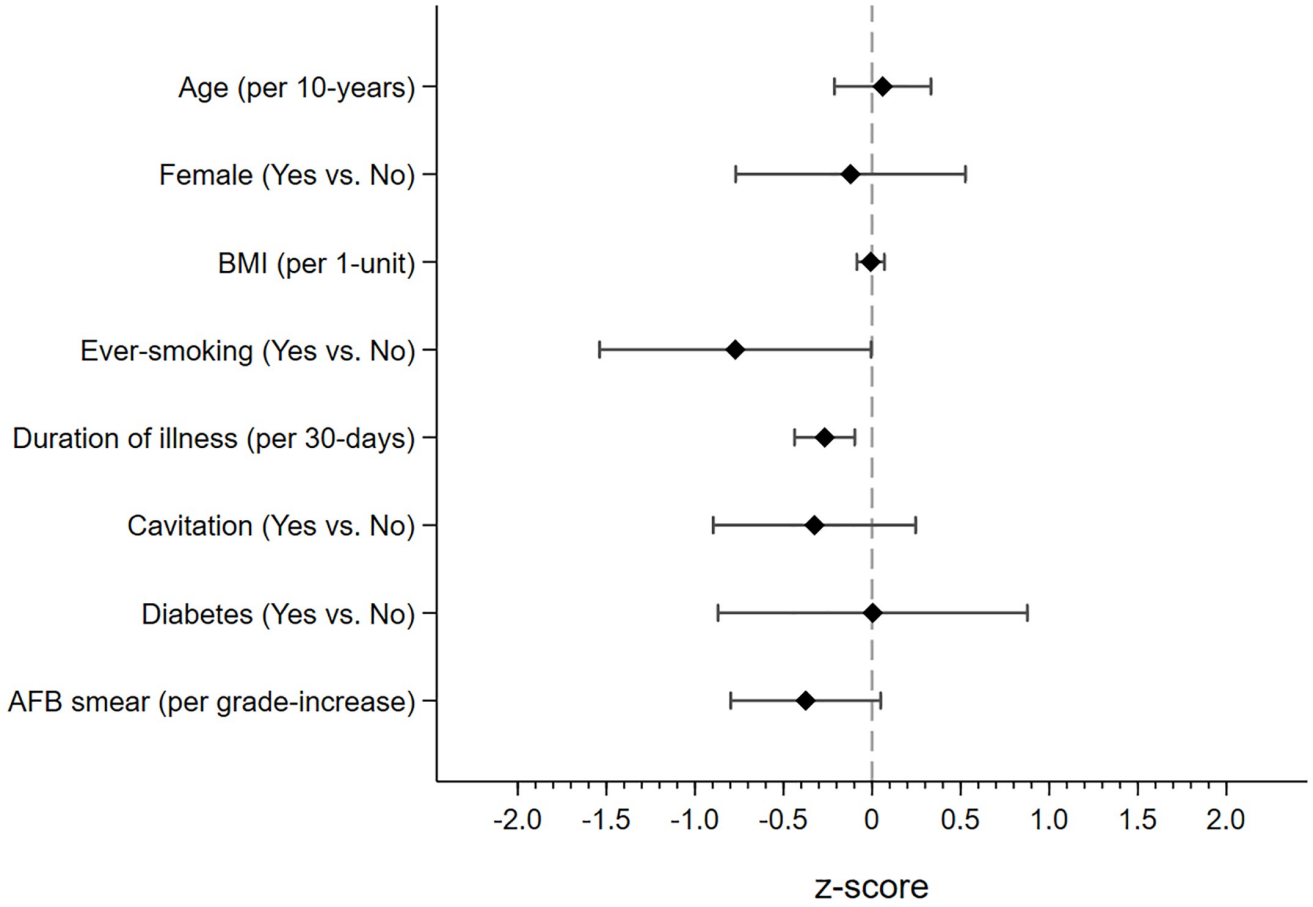
Difference in FEV1/FVC z-score by participant characteristics at treatment initiation. Figure depicts point estimate and accompanying 95% confidence interval for FEV1/FVC standardized by z-scores. Lower z-scores indicate greater degree of airflow obstruction. Regression analysis was adjusted for age, sex, BMI, ever-smoking, duration of illness, cavitation, diabetes and smear grade. BMI—body mass index, AFB—acid fast bacilli.

Higher BMI at treatment initiation was associated with higher FEV1 (0.16 higher z-score per 1kg/m^2^, 95%CI 0.07 to 0.24, p<0.001) and FVC (0.16 higher z-score per 1kg/m^2^, 95%CI 0.07 to 0.26, p<0.001), but not FEV1/FVC, AO or RSP following treatment. Conversely, ever-smoking was associated with lower FEV1/FVC (0.77 lower z-score, 95%CI -1.53 to -0.01, p = 0.04), but not FEV1 and FVC, compared to never-smokers. Longer duration of illness prior to treatment was associated with lower FEV1 (0.15 lower z-score per 30-days, 95%CI -0.34 to 0.02, p = 0.09) and FEV1/FVC (0.26 lower z-score per 30-days, 95%CI -0.43 to -0.09, p = 0.002). After adjusting for potential confounders, duration of illness prior to treatment was associated with higher odds of AO (aOR = 1.32 per 30-days, 95%CI 1.04 to 1.68, p = 0.02). Similarly, higher smear grade at treatment initiation was associated with lower FEV1/FVC (0.37 lower z-score per grade increase, 95%CI -0.79 to 0.04, p = 0.08) and participants with a smear grade of 1+ at treatment initiation were more likely to have AO compared to those with smear negative TB (aOR = 2.38, 95%CI 0.96 to 5.88, p = 0.06). While we did not find an association between sex and lung function, women were more likely to have RSP compared to men (aOR = 3.73, 95%CI 1.51 to 9.17, p = 0.004). Finally, participants with diabetes had lower FVC (1.01 lower z-score, 95%CI -2.05 to 0.02, p = 0.05) and higher odds of RSP (aOR = 4.06, 95%CI 1.14 to 14.43, p = 0.03) relative to those without diabetes. Furthermore, higher HbA1c was associated with higher odds of RSP (aOR = 1.29 per unit HbA1c, 95%CI 1.04 to 1.60, p = 0.02). (Figs 2–4, Table 3).

**Table 3:** Participant characteristics at treatment initiation associated with lung function defects following successful treatment.

Characteristics	AO		RSP	
	aOR (95%CI)	p-value	aOR (95%CI)	p-value
**Age (years)**				
18–29	Ref		Ref	
30–39	0.92 (0.33–2.51)	0.87	1.37 (0.57–3.29)	0.47
≥ 40	1.03 (0.30–3.49)	0.95	0.92 (0.30–2.85)	0.89
**Sex**				
Male	Ref		Ref	
Female	0.56 (0.19–1.58)	0.27	3.73 (1.51–9.17)	0.004
**BMI (kg/m**^**2**^**)**				
>18.5	Ref		Ref	
16–18.5	1.55 (0.58–4.09)	0.37	0.46 (0.19–1.11)	0.09
<16	1.71 (0.57–5.10)	0.33	1.61 (0.60–4.30)	0.34
**Ever-smoking**				
No	Ref		Ref	
Yes	1.50 (0.52–4.38)	0.44	1.10 (0.39–3.08)	0.84
**Duration of illness**				
Per 30-days	1.32 (1.04–1.68)	0.02	0.88 (0.70–1.11)	0.30
**Cavitation**				
No	Ref		Ref	
Yes	1.71 (0.72–4.07)	0.22	0.57 (0.26–1.25)	0.16
**Diabetes**				
No	Ref		Ref	
Yes	0.67 (0.16–2.69)	0.57	4.06 (1.14–14.43)	0.03
**Smear grade**				
Negative	Ref		Ref	
1+	2.38 (0.96–5.88)	0.06	0.42 (0.18–1.00)	0.05
≥2+	2.31 (0.59–8.92)	0.22	0.75 (0.20–2.79)	0.67

AO—airflow obstruction, RSP—restrictive spirometry pattern, aOR—adjusted odds ratio, CI—confidence interval, Ref—reference group. Regression analysis was adjusted for age, sex, BMI, ever-smoking, duration of illness, cavitation, diabetes and smear grade.

The fixed-ratio definition (FEV1/FVC<70%) classified 13 fewer participants with AO. Results from our secondary analysis were consistent with those reported for the LLN definition for AO. (S1 Table).

## Discussion

We found an alarmingly high burden of previously undiagnosed lung function defects (exceeding 75%) that were associated with delays in TB treatment, smear grade at treatment initiation, female sex and diabetes in an Indian cohort of young and predominantly never-smoking adult TB cases who successfully completed treatment. AO and RSP were detected in 24% and 52% participants respectively; over 50% of participants with AO had COPD. While obstruction was predominantly irreversible, 21% of treated TB cases with AO had a clinically meaningful response to short-acting bronchodilators and therefore are likely to benefit from bronchodilator therapy. Our study finding of a disproportionately high burden of AO, a subset of which may respond to bronchodilator therapy, in a population that is typically overlooked during screening for chronic lung diseases due to the absence of conventional risk-factors suggests urgent changes are needed to the current paradigm of TB management which largely ignores follow-up after treatment. We recommend routine screening for chronic lung diseases following TB treatment and linkage to appropriate respiratory health services in all TB cases regardless of their age, smoking exposure and treatment outcomes.

The association between treated TB and chronic lung diseases has been described previously[5, 12]. Studies from North and South America[4, 11, 13], sub-Saharan Africa[28–31] and East Asia have found a high burden of ventilatory defects in participants with a prior history of TB[9, 32, 33]. However, the epidemiology and prevalence of TB and chronic lung diseases likely differs by study setting and similar studies in the Indian context, particularly among individuals with low risk of pre-existing lung disease, are lacking. While the prevalence of AO in the Indian general population is unknown, the prevalence of chronic bronchitis defined by clinical criteria is estimated to be 4.1%[34]. Furthermore, the multi-country BOLD study estimated a 10.1% global prevalence of spirometry-defined COPD in smoking and non-smoking adults aged 40 years and older[35]. We demonstrate that the burden of AO and COPD in new drug-sensitive TB cases who achieve microbiological cure is significantly higher than both global and India-specific estimates from the general population. A recent study in western India reported AO in 87% of treated TB cases[36]. While this estimate is considerably higher than that found in our cohort and elsewhere[11, 37], younger age, lower severity of TB disease and exclusion of prior TB in our study population may explain some of these differences. Banu-Rekha and colleagues reported lung function defects, predominantly a RSP, in 65% of TB cases 14 to 18 years after treatment in southern India[38]. However, given the retrospective nature of this study, advanced age of the study population and, the long duration between TB and lung function assessments, it is likely that physiological processes of lung ageing and exposure to non-TB risk-factors of chronic lung diseases may have contributed to the disease burden. Our study addresses some of these limitations by evaluating the burden and phenotype of lung function defects shortly after microbiological cure in a well characterized prospective cohort of TB cases without drug-resistance or a prior history of TB. Importantly, only 14% (6 of 42) of AO in our study was detected in smokers older than 40 years. The vast majority of AO was detected in young never-smoking adults who are not considered at high-risk for chronic lung diseases and therefore are usually excluded from screening activities. Furthermore, individuals with a history of TB have higher mortality rates than the general population[39–41]. While the precise reasons for this are unclear, chronic lung diseases, especially a RSP may play an important role[42]. Our study highlights the detrimental impact of TB on lung health in high-burden countries, particularly among young adults, and provides evidence for routine screening for chronic lung diseases along with linkage to appropriate respiratory health clinics following treatment of TB cases regardless of their age, smoking exposure and treatment outcomes.

While majority of the AO detected in our study was irreversible, 21% of participants had a positive bronchodilator response suggesting at least partial reversibility of AO. Long acting bronchodilator therapy, individually or in combination with synergistic drugs, is central to the management of COPD[27]. Bronchodilator therapy has been shown to reduce hyperinflation and improve lung function, exercise capacity and quality of life[43, 44]. While the absence of acute bronchodilator responsiveness is indeed a poor predictor for effectiveness of long-acting bronchodilator therapy, individuals with a positive bronchodilator response are likely to receive the greatest therapeutic benefit[45]. Our study found that participants who responded to bronchodilators also tended to have worse respiratory health status and lung function compared to those who did not respond, and may benefit from long-term bronchodilator therapy. Given that pulmonary sequelae of TB may account for nearly 80% of all morbidity and disability associated with the disease[46], clinical trials to evaluate the health benefits of bronchodilator therapy in treated TB cases should be considered.

To our knowledge, our study is the first to demonstrate a significant association between diabetes and lung function impairment in TB cases following treatment completion. We found that treated TB cases with diabetes had lower FVC and higher odds of having RSP compared to those without diabetes after adjusting for potential confounders such as age, sex, BMI and smoking. Furthermore, we found a dose-response relationship between higher HbA1c and higher odds of RSP. Our findings are in contrast to those reported by Lee and colleagues who found a protective effect of diabetes on COPD in a retrospective analysis of health insurance databases[47]. However, this protective effect was attenuated after adjusting for smoking exposure in their study; residual confounding and competing risks may explain some of these differences. Diabetes has previously been associated with COPD, pulmonary fibrosis and lower lung function in individuals without TB[48–51]. Importantly, individuals with diabetes are at increased risk of TB and TB cases are more likely to have diabetes compared to the general population[52–54]. The intersection of these two epidemics, particularly in low- and middle-income countries like India, is likely to pose a serious threat to global lung health. Well-powered studies evaluating pulmonary complications and the underlying immune mechanisms of the TB-diabetes interaction are urgently needed.

Another important finding from our study was the association between delay in treatment initiation and AO. Our findings are consistent with those reported elsewhere[28, 47], reinforcing the importance of early diagnosis and treatment of pulmonary TB which will not only reduce community transmission and improve clinical outcomes, but also prevent pulmonary sequelae in TB. We additionally found that higher BMI at treatment initiation was associated with better lung function, while participants with higher smear grade at baseline had lower FEV1/FVC ratios and were more likely to have AO following treatment. Higher smear grade may indicate greater severity of TB disease which has previously been shown to correlate with the degree of pulmonary sequelae[55, 56]. Low BMI may reflect greater severity of TB disease, chronic malnutrition, or both[57], and has been independently associated with poor lung function[58, 59]. Malnutrition is strongly linked to TB and its clinical prognosis[60]. Monitoring TB cases with low BMI for pulmonary complications and sequelae in addition to culture and smear conversion should be considered. While formal education was associated with a marginally lower FEV1, we did not find similar associations with AO or RSP. Education may be a surrogate of socio-economic status, an independent risk factor for low lung function[61]. Finally, we found that women were significantly more likely to have RSP compared to men. While the precise reasons for this are unclear, inherent gender differences or exposure to household biomass fuels in women may partly explain our study findings[62]. Future studies should actively consider the impact of biomass fuel exposure on respiratory health of women with TB.

Our study has some limitations. Pre-treatment spirometry was not available. Although a history of chronic lung diseases was an exclusion criterion for our study, undiagnosed lung impairment prior to TB onset may have biased our results. However, our study population was comprised predominantly of young never-smokers without a prior history of TB and the burden of pre-existing chronic lung diseases in this population is likely to be low. Importantly, none of the participants with chronic lung diseases were aware of their disease status prior to the study. Regardless of the temporality with onset of TB, screening and referral following treatment completion can detect previously undiagnosed chronic lung diseases in this high-risk population. Another potential limitation of our study was the use of reference equations derived from non-Indian populations which may have overestimated the burden of lung function defects. However, in the absence of valid reference equations from Indian populations explicitly accounting for the dynamic changes in lung function in early adulthood[63, 64], the GLI mixed-ethnicity estimates remain a valid choice.

Despite these limitations, our study demonstrated a high burden of undiagnosed lung function defects in a well characterized cohort of young and predominantly never-smoking adult TB cases who successfully completed treatment in India. We recommend linkages between TB control programs and respiratory health clinics for the timely diagnosis and management of post-TB pulmonary sequelae, regardless of microbiological outcomes or the presence of conventional risk factors such as older age and smoking. We additionally recommend well-powered studies to evaluate the immune mechanisms of lung injury in TB cases with diabetes, and clinical trials for evaluating the long-term benefit of bronchodilator therapy in treated TB cases with AO.

## Supporting information

S1 Table Participant characteristics at treatment initiation associated with airflow obstruction, classified by the fixed-ratio definition, despite successful TB treatment. aOR—adjusted odds ratio, CI—confidence interval, Ref—reference group. Regression analysis was adjusted for age, sex, BMI, ever-smoking, duration of illness, cavitation, diabetes and smear grade.
(DOCX)
