# Correction: Assessment of lung function in successfully treated tuberculosis reveals high burden of ventilatory defects and COPD

**DOI:** 10.1371/journal.pone.0226389

**Published:** 2019-12-05

**Authors:** Akshay N. Gupte, Mandar Paradkar, Sriram Selvaraju, Kannan Thiruvengadam, Shri Vijay Bala Yogendra Shivakumar, Krithikaa Sekar, Srinivasa Marinaik, Ayesha Momin, Archana Gaikwad, Premkumar Natrajan, Munivardhan Prithivi, Gomathy Shivaramakrishnan, Neeta Pradhan, Rewa Kohli, Swapnil Raskar, Divyashri Jain, Rani Velu, Bharath Karthavarayan, Rahul Lokhande, Nishi Suryavanshi, Nikhil Gupte, Lakshmi Murali, Sundeep Salvi, William Checkley, Jonathan Golub, Robert Bollinger, Vidya Mave, Chandrasekaran Padmapriyadarasini, Amita Gupta

The Data Availability statement for this paper is incorrect. The correct statement is: The minimal anonymized dataset necessary to replicate this study’s findings are uploaded to Dryad (DOI: 10.5061/dryad.1340q4t), and can be downloaded here: https://datadryad.org/stash/share/G2gYDco41fzjgSe1MS07nK_WTzCNBlLmGvrH758QWss.

## References

[1] Gupte AN, Paradkar M, Selvaraju S, Thiruvengadam K, Shivakumar SVBY, Sekar K, et al (2019) Assessment of lung function in successfully treated tuberculosis reveals high burden of ventilatory defects and COPD. PLoS ONE 14(5): e0217289 10.1371/journal.pone.0217289 31120971 PMC6532904

