# Supplementary material for: Assessment of lung function in successfully treated tuberculosis reveals high burden of ventilatory defects and COPD
Source: PLoS One. 2019 May 23;14(5):e0217289. doi: 10.1371/journal.pone.0217289 (PMC6532904; doi:10.1371/journal.pone.0217289)
Supplement: S1 Table — aOR—adjusted odds ratio, CI—confidence interval, Ref—reference group. Regression analysis was adjusted for age, sex, BMI, ever-smoking, duration of illness, cavitation, diabetes and smear grade. (DOCX) [file pone.0217289.s001.docx]

| **Characteristics** | **FEV1/FVC<70%** | |
| --- | --- | --- |
|  | **aOR (95%CI)** | **p-value** |
| **Age (years)** |  |  |
| 18-29 | Ref |  |
| 30-39 | 1.15 (0.35-3.76) | 0.81 |
| > 40 | 2.79 (0.70-11.00) | 0.14 |
| **Sex** |  |  |
| Male | Ref |  |
| Female | 1.27 (0.38-4.24) | 0.69 |
| **BMI (kg/m^2^)** |  |  |
| >18.5 | Ref |  |
| 16-18.5 | 1.53 (0.50-4.66) | 0.45 |
| <16 | 1.51 (0.43-5.29) | 0.51 |
| **Ever-smoking** |  |  |
| No | Ref |  |
| Yes | 2.67 (0.80-8.85) | 0.09 |
| **Duration of illness** |  |  |
| Per 30-days | 1.44 (1.12-1.87) | 0.005 |
| **Cavitation** |  |  |
| No | Ref |  |
| Yes | 1.21 (0.45-3.27) | 0.69 |
| **Diabetes** |  |  |
| No | Ref |  |
| Yes | 0.96 (0.21-4.21) | 0.96 |
| **Smear grade** |  |  |
| Negative | Ref |  |
| 1+ | 1.02 (0.35-2.95) | 0.96 |
| >2+ | 1.98 (0.48-8.15) | 0.33 |
